# External magnetic field-induced circularly polarized luminescence and electroluminescence from optically inactive thermally activated delayed fluorescence material 4CzIPN

**DOI:** 10.3389/fchem.2023.1281168

**Published:** 2023-10-20

**Authors:** Takumi Kuroda, Maho Kitahara, Shigeyuki Yagi, Yoshitane Imai

**Affiliations:** ^1^ Department of Applied Chemistry, Faculty of Science and Engineering, Kindai University, Higashi-osaka, Japan; ^2^ Department of Applied Chemistry, Graduate School of Engineering, Osaka Metropolitan University, Osaka, Japan

**Keywords:** chiral, circularly polarized organic light-emitting diode, 4CzIPN, electroluminescence, magnetic circular dichroism, magnetic circularly polarized luminescence, MCP-OLED device, thermally activated delayed fluorescence

## Abstract

An achiral optically inactive organic luminophore, 4CzIPN, exhibits circularly polarized thermally activated delayed fluorescence when photoexcited under an external magnetic field. By embedding this luminophore in an active emission layer, an external-magnetic-field-induced circularly polarized electroluminescent device is developed in this study. The Faraday geometry of the applied magnetic field completely controls the direction of rotation of 4CzIPN-derived circularly polarized luminescence and electroluminescence.

## 1 Introduction

Many chiral light-emitting materials that exhibit circularly polarized luminescence (CPL) upon unpolarized photoexcitation have, in recent years, been discovered. These luminescent materials have optical properties of anisotropy factor (*g*_CPL_) and high quantum yield (*Ф*_PL_). Therefore, they have been used in circularly polarized light-emitting diodes (CP-LEDs) (Peeters et al., 1997; Zinna et al., 2015; Brandt et al., 2016; Zinna et al., 2017; Li et al., 2018; Frédéric et al., 2020; Jiang et al., 2020; Frédéric et al., 2021; Xie et al., 2021; Yan et al., 2021; Hara et al., 2022). Chiral organic luminophores with sharp CPL bandshapes and high anisotropy factors over a wide range of wavelengths are efficient for use in high-performance CPEL devices. However, obtaining enantiomerically pure luminescent isomers through green and economical methods is difficult. Therefore, it is very important to develop new approaches to the achievement of CPL and circularly polarized electroluminescence (CPEL). CPL and CPEL have various applications, including energy-efficient three-dimensional displays, advanced security tags, and LED-based plant growth control, and new industries using this special polarized light are expected to emerge.

Recently, thermally activated delayed fluorescence (TADF) materials have attracted attention as a third-generation luminescent material with excellent energy-conversion efficiency. This is because the small energy gap between singlet and triplet excited states facilitates endothermic conversion of the triplet to singlet exciton via an inverse intersystem crossing (Endo et al., 2009; Endo et al., 2011; Hirata et al., 2015; Hatakeyama et al., 2016; Guo et al., 2017; Spuling et al., 2018; Congrave et al., 2019). In more detail, the electric current excitation of a luminescent material, as in OLED, gives rise to the spin-statistic generation of singlet and triplet excited states in a ratio of 1:3. According to this spin statics theory, OLEDs comprising conventional fluorescent materials achieve a maximum internal quantum efficiency (IQE) of 25%. Conversely, OLEDs employing TADF emitters can utilize prompt fluorescent emission (i.e., emission from the first generated singlet excited state) as well as delayed fluorescent emission from the singlet excited state generated afterward through thermal triplet-to-singlet up-conversion. Consequently, TADF-based electroluminescent devices utilize whole excitons to achieve extremely high electricity-to-light conversion efficiency (i.e., internal quantum efficiency), theoretically as high as 100% (Uoyama et al., 2012; Zhang et al., 2012; Tao et al., 2014; Kaji et al., 2015; Liu et al., 2018; Kondo et al., 2019). TADF luminophores that exhibit CPL (Zhang et al., 2018; Sharma et al., 2019; Yang et al., 2020; Frédéric et al., 2021) and circularly polarized organic light-emitting diodes (CP-OLEDs) using chiral TADF luminophores that exhibit CPEL (Feuillastre et al., 2016; Wang et al., 2019; Frédéric et al., 2020; Frédéric et al., 2021; Xie et al., 2021) have also attracted considerable attention.

However, CPL materials and CP-OLEDs using TADF also require optical isomers with a high enantiomeric purity. Therefore, new and innovative approaches are required to more conveniently realize CPL and CPEL from TADF materials. One solution to this challenge is to utilize the magnetic circular dichroism (MCD) theory proposed by Riehl and Richardson (Riehl and Richardson, 1977; Ghidinelli et al., 2021) to induce CPL because MCPL can be viewed as the reverse process of MCD. According to this theory, as a versatile physical bias, external static magnetic fields can induce chiral spectral signals from both ground and photoexcited states in many achiral and racemic optically inactive organic, organometallic, and inorganic luminescent materials (Rikken and Raupach, 1997; Valiev et al., 2014; Knowles et al., 2015; Wu et al., 2017; Ivchenko, 2018; Ghidinelli et al., 2020; Imai, 2020; Imai, 2021; Kitahara et al., 2021; Zhang et al., 2021; Hara et al., 2022).

Pyrene-based chiral organic luminescent materials exhibit CPL. We recently found that an achiral optically inactive pyrene luminescent molecule exhibits external-magnetic-field-driven CPL (MCPL) when photoexcited under a 1.6 T external magnetic field (Kaji et al., 2020). Notably, the signs of these MCPL spectra could be completely controlled by modulating the Faraday configuration of the applied external magnetic field direction [N→S (N-up) and S→N (S-up)]. In the Faraday configuration here, the direction of the light is parallel to that of the magnetic field, and N-up and S-up represent the parallel and antiparallel configurations of the longitudinal magnetic field, respectively, concerning the unpolarized incoming light.

**FIGURE 1:**
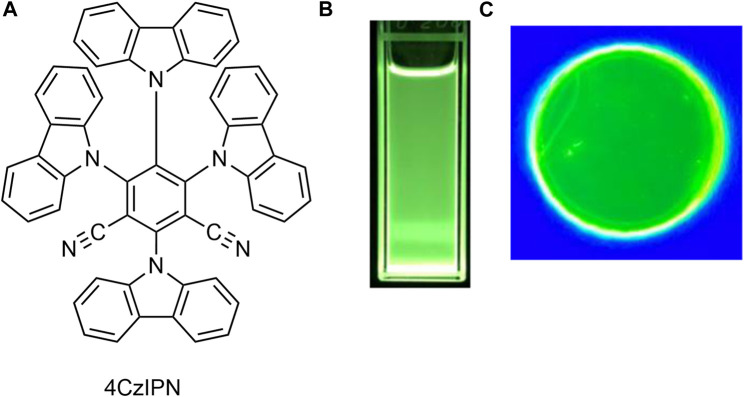
Molecular structure of **(A)** the achiral TADF luminophore, 4CzIPN, and photographs of emitting samples of 4CzIPN in **(B)** deoxygenated CHCl_3_ and **(C)** PMMA film under 365 nm light.

In this work, we successfully generated MCPL by photoexciting TADF-active 1,2,3,5-tetrakis(carbazol-9-yl)-4,6-dicyanobenzene (or 2,4,5,6-tetrakis(9*H*-carbazol-9-yl)isophthalonitrile (4CzIPN) in solution and poly(methyl methacrylate) (PMMA)-film states under a 1.7 T external magnetic field (Figure 1). Furthermore, a new magnetic circularly polarized organic light-emitting diode (MCP-OLED) incorporating 4CzIPN as an emitting dopant has been successfully developed. By applying a 1.7 T external magnetic field and an appropriate voltage to the developed MCP-OLED, magnetic circularly polarized electroluminescence (MCPEL) could be emitted. Furthermore, by changing the Faraday configuration of the applied external magnetic field from N-up to S-up, the transition sign between the MCPL and MCPEL processes was completely controlled.

## 2 Methods

### 2.1 Production of the MCP-OLED device

4CzIPN was purchased from Sigma-Aldrich Japan (Tokyo, Japan). Indium tin oxide (ITO) substrates were ultrasonically cleaned in solution using ultrapure water and an alkaline detergent. The cleaned substrate surface was wetted with isopropyl alcohol vapor and treated with ultraviolet ozone. All organic thin films and Al were then deposited on the substrate using the vacuum evaporation method. During deposition, the temperature and deposition rate of the deposition source were precisely controlled to adjust the doping concentration of the luminescent active layer. Specifically, organic layers, such as HATCN, NPD, TCTA, DBT-TRZ, and Liq, were thermally evaporated under 10^−5^ Pa at a deposition rate of 0.1–2.0 Å/s. For the co-evaporated layers of the luminescent active layer (EML and nBphen:Liq), the constituent ratio was adjusted by tuning the vacuum-deposition rates of each material. The deposition system was connected to a glove box via a load lock chamber, and oxygen and moisture concentrations were controlled to <10 ppm. Thus, during device fabrication, the substrate with layer-by-layer thin films of the constituent materials was almost shielded from oxygen and moisture. After deposition, the devices were completely sealed in a glass container to protect them from the external atmosphere.

### 2.2 MCD and ultraviolet (UV)–visible absorption spectroscopy

The MCD and ultraviolet (UV)–visible absorption spectra of 4CzIPN in a deoxygenated CHCl_3_ solution and a PMMA doped film were recorded using a JASCO J-1700 spectropolarimeter (Hachioji, Tokyo, Japan) at 25°C. An external magnetic field of 1.7 T was applied using a JASCO PM-491 permanent magnet. The bandwidth was set to 2 nm, the scan rate was 100 nm/min, and the spectrum was measured for single acquisition. The optical path length for the solution-state measurement was 5 mm. The PMMA films incorporated with 4CzIPN for MCD and UV–visible absorption spectroscopy were prepared using the same method described for preparing samples for MCPL and magnetic photoluminescence (MPL) evaluations.

### 2.3 MCPL and MPL spectroscopy

The MCPL and MPL spectra of 4CzIPN were recorded at 25°C using a JASCO CPL-300 spectrofluoropolarimeter (Hachioji, Tokyo, Japan). A 1.7 T external magnetic field was applied using a JASCO PM-491 permanent magnet. Spectra were recorded under unpolarized monochromatic UV light (380 nm) excitation with a bandwidth of 10 nm and a scattering angle of 0°. The optical path length for spectroscopic measurements in solution was 5 mm. The 4CzIPN-doped PMMA films were prepared by the spin-coating method using a Mikasa spin-coater (MS-A100) (Tokyo, Japan) rotating at 3,000 rpm. A mixture of 7.9 mg of 4CzIPN and 10 mg of PMMA was dissolved in CHCl_3_ (1.0 mL), and a portion of the solution (0.8 mL) was used for spin-coating to prepare a 4CzIPN-doped PMMA film.

### 2.4 Evaluation of electroluminescence (EL), magnetic electroluminescence (MEL), and MCPEL

MCPEL and unpolarized MEL spectra of the MCP-OLEDs were acquired at 25°C using a JASCO CPL-300 spectrofluoropolarimeter (Hachioji, Tokyo, Japan). A 1.7 T external magnetic field was applied using a JASCO PM-491 permanent magnet. The emission bandwidth was 10 nm. The brightness of emission was recorded using a BM-9 luminance meter (TOPCON TECHNOHOUSE CORPORATION, Japan). The external quantum efficiency (EQE) of the fabricated device was evaluated at 25 °C using a Hamamatsu Photonics C-9920-11 organic EL device evaluating system (Hamamatsu, Japan).

## 3 Results and discussion

### 3.1 MCD and MCPL properties of 4CzIPN

**FIGURE 2:**
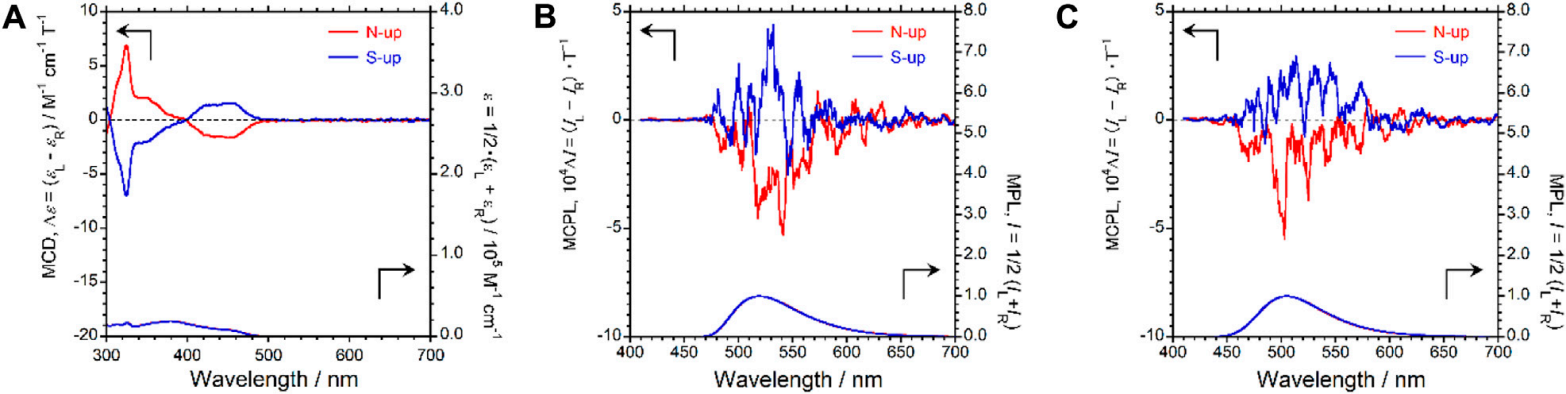
MCD (top panel) and UV–visible absorption spectra (bottom panel) of 4CzIPN in **(A)** deoxygenated CHCl_3_ (conc = 1.0 × 10^−4^ M). MCPL (top) and MPL spectra (bottom) of 4CzIPN in **(B)** deoxygenated CHCl_3_ (conc = 1.0 × 10^−3^ M) and **(C)** PMMA-film state under magnetic fields (1.7 T). Red and blue lines show N-up and S-up Faraday geometries, respectively.

4CzIPN is an achiral optically inactive compound; neither circular dichroism (CD) nor CPL was observed under ambient conditions. Therefore, we investigated the magnetic-field-induced CD and CPL (MCD and MCPL) properties of 4CzIPN dissolved in deoxygenated CHCl_3_ by applying a 1.7 T external magnetic field. The experimental MCD, MCPL, and MPL spectra obtained under the applied magnetic field are shown in Figures 2A, B, respectively. Although 4CzIPN is an optically inactive luminophore, the MCD cotton effects were observed at 324, 350, 429, and 454 nm. As expected, a set of mirror-symmetric MCPL signals corresponding to the MPL signal was observed at 525 nm. Notably, when the direction of the applied external magnetic field was reversed (N-up or S-up), the signs of the MCD (blue line for S-up and red line for N-up) and MCPL (blue line for S-up and red line for N-up) spectra were also reversed.

The magnitude of circular polarization in the ground state is expressed by the MCD anisotropy factor given by the equation:

$$g_{\text{MCD}}=\frac{\left({Abs}_{L}-{Abs}_{R}\right)}{\left[\left({Abs}_{L}+{Abs}_{R}\right)\;/\;2\right]}$$

In this equation, Abs_L_ and Abs_R_ represent the absorbances of left- and right-rotating circularly polarized light, respectively, under an external magnetic field. In contrast, to quantitatively evaluate the magnitude of MCPL, the MCPL anisotropy coefficient, given by

$$g_{\text{MCPL}}=\frac{\left(I_{L}-I_{R}\right)}{\left[\left(I_{L}+I_{R}\right)/2\right]}$$

was investigated. In this equation, *I*_L_ and *I*_R_ are the intensities of the left- and right-rotating MCPL, respectively, observed upon unpolarized UV light excitation under a magnetic field. In other words, a positive sign in the MCPL spectrum indicates left-handed circularly polarized emission, whereas a negative sign indicates right-handed circularly polarized emission. The absolute values of the anisotropy factors of 4CzIPN, |*g*_MCD_| and |*g*_MCPL_|, in the CHCl_3_ solution were 2.1 × 10^−4^ (T^−1^) at the longest magnetic circular dichroic absorption wavelength (*λ*_MCD_) of 454 nm and 4.6 × 10^−4^ (T^−1^) at the MCPL wavelength (*λ*_MCPL_) of 525 nm, respectively.

When considering the use of 4CzIPN as an emitting dopant in an MCPEL device, it is important to study its chiroptical properties in a solid film state rather than in a solution state. This is because luminescence phenomena in a solution and solid matrix often differ significantly, and studying the luminescence behavior in solid organic media, such as PMMA polymers, is important for predicting the field emission behavior of OLEDs. Therefore, we doped 4CzIPN in a PMMA film and investigated the MCPL properties of the resultant film. When the 4CzIPN-incorporated PMMA film was photoexcited under a 1.7 T external magnetic field, a set of mirror-image MCPL spectra was observed at 508 nm, the spectral shapes of which correspond to that of the MPL spectrum, as shown in Figure 2C. Notably, as observed in the solution state, the sign of the MCPL spectrum (blue and red lines for S-up and N-up, respectively) could be completely reversed by changing the direction of the applied magnetic field from N-up to S-up. The absolute value of the anisotropy factor |*g*_MCPL_| of MCPL exhibited by the 4CzIPN-doped PMMA film was 4.3 × 10^−4^ (T^−1^).

As described, although 4CzIPN is an optically inactive luminescent material, MCPL was successfully observed in both the CHCl_3_ solution state and the PMMA-film state upon photoexcitation under an external magnetic field. A slight increase in the anisotropy factor was observed in the excited state in relation to that in the ground state. Conversely, no significant difference was observed in the anisotropy factors of the excited states of the luminophore in the CHCl_3_ solution and PMMA-film states. This result indicates that the effect observed under the external magnetic field does not depend on the external environment of the luminophore, i.e., fluidic CHCl_3_ or solid PMMA.

### 3.2 Evaluation of device properties

The OLED using 4CzIPN as an emitting dopant in the active emission layer was fabricated and investigated for its MCPEL properties. The direction of the applied external magnetic field was changed to N-up and S-up, and the MCPEL profiles were obtained at room temperature.

**FIGURE 3:**
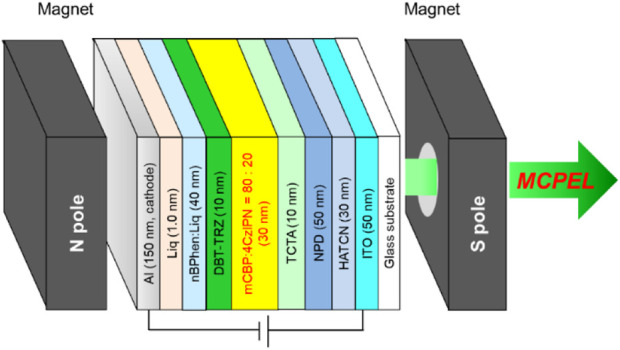
Configuration diagram of the MCP-OLED system composed of OLED structure and small interchangeable N- and S-pole magnets.

**FIGURE 4:**
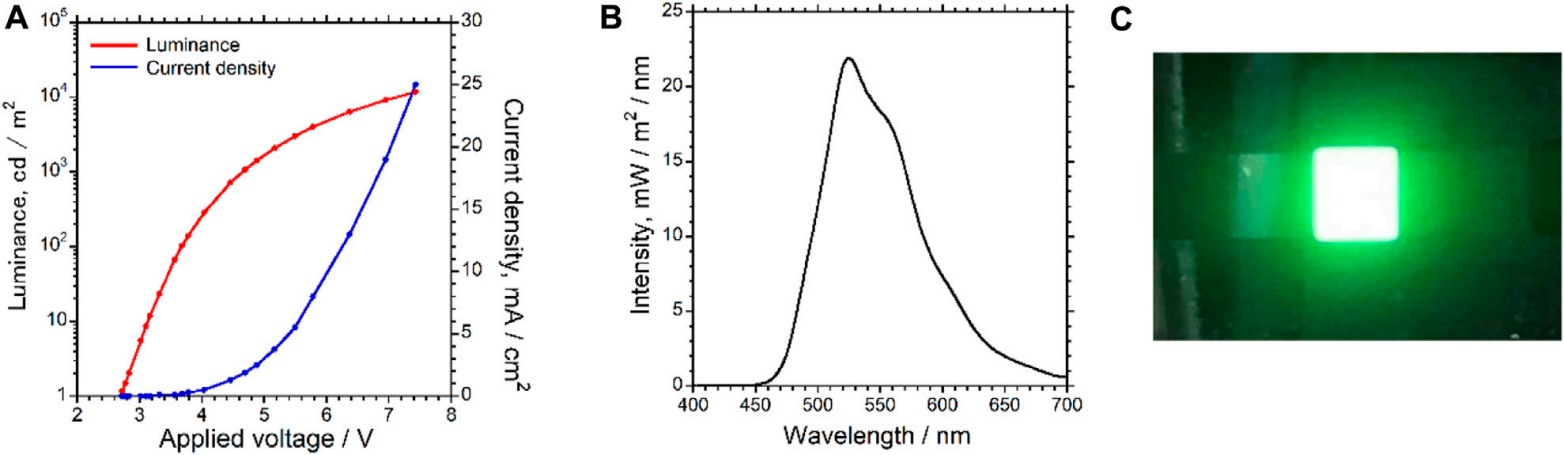
**(A)** Current density vs. voltage vs. luminance (*J*–*V*–*L*) profiles and **(B)** electroluminescence (EL) spectrum for the 4CzIPN-based device. **(C)** Photograph of the 4CzIPN-based device upon application of an electric voltage of 8 V.

The multi-stack structure of the fabricated 4CzIPN-based device is shown in Figure 3. It was structured as follows: ITO (anode, 50 nm)/HATCN (30 nm)/NPD (50 nm)/TCTA (10 nm)/emission layer (EML, 30 nm)/DBT-TRZ (10 nm)/nBPhen:Liq (40 nm)/Liq (1.0 nm)/Al (cathode, 150 nm). In this, HATCN, NPD, TCTA, DBT-TRZ, nBPhen:Liq and Liq are abbreviations for 1,4,5,8,9,11-hexaazatriphenylenehexacarbonitrile, *N*,*N*′-di-1-naphthyl-*N*,*N*′-diphenylbenzidine, 4,4′,4″-tri(9-carbazolyl)triphenylamine, 2-(3'-(dibenzo[*b*,*d*]thiophen-4-yl)-[1,1′-biphenyl]-3-yl)-4,6-diphenyl-1,3,5-triazine, 2,9-bis(naphthalen-2-yl)-4,7-diphenyl-1,10-phenanthroline:lithium quinolin-8-olate, and lithium quinolin-8-olate, respectively. The EML consists of 3,3′-di(9*H*-carbazol-9-yl)-1,1′-biphenyl (mCBP) and 4CzIPN at 80/20 w/w. HATCN is a charge-generation and hole-injection layer, facilitating the generation-injection of positively charged carriers (holes) to the organic layers from the ITO anode. NPD and TCTA are hole-transporting layers with p-type semiconducting characteristics, which receive holes from the neighboring hole-injection layer and inject them into the EML. DBT-TRZ is a hole-blocking layer with a deep highest occupied molecular orbital (HOMO) of 6.2 eV below the vacuum level (Saito et al., 2021). As the host matrix of the EML is mCBP with p-type semiconducting characters, the holes are blocked and pooled at the EML/DBT-TRZ interface. nBPhen:Liq is an electron-transporting layer with n-type semiconducting characteristics, facilitating the injection of electrons to the EML via the DBT-TRZ layer. Liq is an electron-injection layer to assist the smooth insertion of electrons from the Al cathode to the electron-transporting layer. This type of multi-stacked device structure allows us to achieve efficient hole‒electron recombination at the EML through the well-ordered, unidirectional injection of holes and electrons from the anode and cathode, respectively, achieving a high luminous efficiency. The current density–voltage–luminance (*J*–*V*–*L*) curves, the EL spectrum, and the related parameters were recorded; the corresponding results are shown in Figure 4 and summarized in Table 1.

**TABLE 1:** Performance of the 4CzIPN-based device.

*λ*_*EL*_ (nm)	*V*_on_^[a]^ (V)	*L*_max_^[b]^ (cd·m^−2^) [@V]	*η*_j, max_^[b]^ (cd·A^−1^) [@V]	CIE (*x*, *y*)^[c]^	EQE_max_^[d]^ (%) [@V]
524	2.7	11770 [7.4]	57.44 [4.5]	(0.34, 0.59)	15.5 [6.5]

[a] Turn-on voltage at which a luminance of 1 cd m^−2^ or higher was obtained. [b] Maximum values of luminance (*L*) and current efficiency (*η*_j_). Corresponding operating voltages are provided in parentheses. [c] Commission Internationale de L’Eclairage (CIE) chromaticity coordinates. [d] Maximum value of the EQE.

As seen in the *J*–*V*–*L* curves (Figure 4A), the fabricated device exhibited a semiconductive behavior and started to emit green EL upon applying a voltage of 2.7 V, as seen in Figures 4B, C. When a voltage of 7.4 V was applied to this device, the maximum luminance (*L*_max_) was 11770 cd m^−2^ at 7.4 V, indicating that the device performance was suitable for MEL and MCPEL data acquisition under an externally applied magnetic field. Figure 4B shows that the EL spectrum was almost comparable to the PL spectrum of 4CzIPN, in which no emission from the host material mCBP was observed. Thus, charge carrier and/or exciton transfer from mCBP to 4CzIPN occurred efficiently. The Commission Internationale de L'Eclairage chromaticity coordinates at the *L*_max_ were to be (x, y) (0.34, 0.59). Furthermore, the device parameters shown in Table 1 suggest that the present device exhibits highly efficient device performance. Notably, the EQE of the device (maximum value: 15.5% at 6.5 V) was considerably higher than the theoretical limit of EQE for conventional fluorescent OLEDs (∼5%) (Uoyama et al., 2012), which is a characteristic of TADF-based OLEDs. This result strongly suggests that the observed EL consisted of prompt and delayed fluorescence emissions from 4CzIPN. Therefore, the fabricated 4CzIPN-containing OLED device should be a suitable candidate for TADF-based MCPEL devices.

**FIGURE 5:**
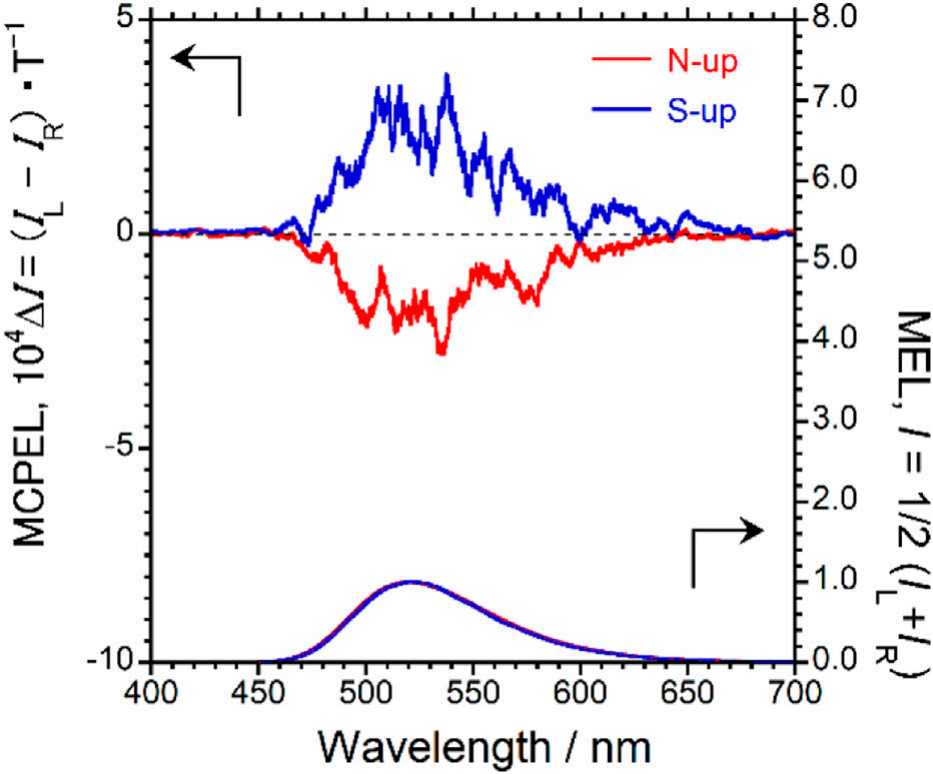
MCPEL (top panel) and MEL (bottom panel) spectra of the 4CzIPN-based device under magnetic fields (1.7 T). Red and blue lines show N-up and S-up Faraday geometry, respectively.

Next, under a 1.7 T magnetic field, the MCPEL properties of the 4CzIPN-based device were studied at 25°C. In the measurement of the MCPEL and MEL characteristics of this device, the applied voltage was fixed at 8 V. Figure 5 shows the obtained MCPEL and MEL spectra. As expected, we succeeded in obtaining green MCPEL from the 4CzIPN-based device although it is composed entirely of achiral and optically inactive components. Furthermore, mirror-image MCPEL spectra were recorded upon alternating the Faraday configuration of the N-up/S-up external magnetic fields. These results prove that the direction of rotation of MCPEL exhibited by the device can be easily controlled by changing the direction of the applied external magnetic field.

The MCPEL and MEL spectra of the 4CzIPN-based device were similar to the MCPL and MPL spectra of 4CzIPN in the PMMA-film state, respectively. It is very common that the EL spectra of OLEDs and the PL spectra of emitting materials used are similar. When comparing the MCPEL spectra of the device to the MCPL spectra of the emitting material, the signs of the MCPEL and MCPL spectra were observed to be negative (−) in the N-up geometry, whereas they were positive (+) in the S-up geometry. In other words, the direction of rotation of MCPEL exhibited by this device was the same as that of MCPL exhibited by the same 4CzIPN luminescent material in CHCl_3_ solution and under PMMA-film conditions. This comparison shows that this generalization also applies to the present 4CzIPN-based device.

The performance of the devices that exhibit MCPEL upon the application of an external magnetic field is quantitatively evaluated using the anisotropy factor,

$$g_{MCPEL}=\frac{\left(I_{L}-I_{R}\right)}{\left[\left(I_{L}+I_{R}\right)/2\right]}$$

similar to the performance evaluation of CPEL. *I*_L_ and *I*_R_ are the amplitude intensities of the left- and right-handed MCPEL, respectively. As with the MCPL spectrum, positive sign in the MCPEL spectrum indicate leftward circularly polarized electroluminescence, whereas negative sign indicate rightward circularly polarized electroluminescence. For this 4CzIPN-based device, the anisotropy factor |*g*_MCPEL_| was 3.7 × 10^−4^ (T^−1^) at 537 nm. In general, the anisotropy factor of the CP-OLED devices decreases when multiple interfaces of the multilayer device interfere unfavorably with circular polarization. However, the |*g*_MCPEL_| value of this 4CzIPN-based was almost identical to the corresponding values obtained from a CHCl_3_ solution (4.6 × 10^−4^ (T^−1^) at 525 nm) or a PMMA film (4.3 × 10^−4^ (T^−1^) at 508 nm). This indicates that, in this 4CzIPN-based system, the circular polarization of the photoexcited luminescence and the electroluminescence are the same.

These results indicate that efficient MCPEL can be observed under a 1.7 T magnetic field and at an appropriate voltage for OLEDs containing optically inert 4CzIPN emitters. This study, using TADF-active 4CzIPN, is the first example of MCPEL generated from TADF-OLED and shows that it is possible to promote the development of highly functional MCPEL devices using optically inactive TADF luminescent materials with various functionalities.

## 4 Conclusion

Optically inactive 4CzIPN with TADF properties does not exhibit CPL under conventional photoexcitation conditions. However, upon applying a 1.7 T external magnetic field, it exhibited MCPL. Furthermore, an MCP-OLED using 4CzIPN as an emitting dopant exhibited green MCPEL under a 1.7 T magnetic field. The chiroptical sign of MCPEL exhibited by this device could be accurately controlled by modulating the Faraday geometry of the magnetic field (N-up or S-up), which was observed with the chiroptical sign of the MCPL of 4CzIPN. This external-magnetic-field-driven MCP-OLED device is expected to have promising application potential in CPEL and spin-LED devices.

## Figures and Tables

**Table:** 

**4CzIPN**	1,2,3,5-tetrakis (carbazol-9-yl)-4,6-dicyanobenzene (or 2,4,5,6-tetrakis (9*H*-carbazol-9-yl)isophthalonitrile)
**MCPEL**	Magnetic-field-driven circularly polarized electroluminescence
**MCPL**	Magnetic-field-driven circularly polarized luminescence
**MPL**	Magnetic photoluminescence
**OLED**	Organic light-emitting diode
**PMMA film**	Poly (methyl methacrylate) film
**TADF**	Thermally activated delayed fluorescence
**CD**	Circular dichroism

## Data Availability

The original contributions presented in the study are included in the article/Supplementary Material, further inquiries can be directed to the corresponding author.
